# Increase in the Use of Alcohol and Other Substances in Adolescents During and After the COVID-19 Pandemic

**DOI:** 10.1192/j.eurpsy.2022.58

**Published:** 2022-09-01

**Authors:** G. Dom

**Affiliations:** University of Antwerp, Psychiatry, Boechout, Belgium

## Abstract

The COVID-19 pandemic and its associated restriction measures have a great impact on patterns of substance use throughout the general population. Interestingly, in contrast to initial expectations on an overall negative impact, data of large surveys show a differentiated picture of impact. A recent systematic review (Roberts et al., 221) showed mixed tendencies towards increased alcohol consumption, contrasting with a clear trend towards the use of other psychoactive (illicit drugs and prescription drugs) substances. patterns of use before COVID-19 (e.g. habit of drinking at home) and availability (illicit drug traffic changes) are among the significant factors. In addition, mental health factors (depression, anxiety) and social isolation are common correlates with substance use. These factors may be of specific importance impacting substance use in adolescents during the COVID-19 pandemic. Exemplary is an increase in the use of sleeping medication among Belgian adolescents and young adults. In the current presentation data of national (Belgium) surveys on substance use in adolescents will be presented and discussed within the context of findings in international surveys.

**Disclosure:**

No significant relationships.

